# Gut microbiome differences after vaginal birth in relation to rupture of membranes at term: a prospective longitudinal cohort study of twins

**DOI:** 10.1007/s00431-025-06336-w

**Published:** 2025-07-30

**Authors:** Marcos Javier Cuerva, Irene Bartha, Esperanza Escribano, Guillermo Chueca, Marta Perez de Aguado, Irene Espinosa-Martos, Sergio Esteban, Maria De La Calle, Esther Jimenez, Jose Luis Bartha

**Affiliations:** 1 Department of Obstetrics, Hospital Universitario La Paz, Paseo La Castellana, 261, 28046 Spain Madrid,; 2 School of Medicine, Universidad Autónoma de Madrid, Madrid, Spain; 3 Maternal and Fetal Medicine Research Group, Matenal, Infant and Adolescent Medicine Area of IdiPaz (Foundation for BioMedicine Research), Madrid, Spain; 4 Department of Women and Children’s Health (Paediatric Allergy, School of Life Course Sciences, Faculty of Life Sciences and Medicine, King’s College London and Guy’s and St Thomas’ NHS Foundation Trust, London, UK; 5 Department of Neonatology, Hospital Universitario La Paz, Madrid, Spain; 6 Probisearch SLU, Tres Cantos, Spain; 7 Spanish Network in Maternal, Neonatal, Child and Developmental Health Research (RICORS-SAMID, RD24/0013/0018), Instituto de Salud Carlos III, Madrid, Spain

**Keywords:** Bifidobacterium, Childbirth, Gut microbiome, Rupture of membranes, Twins, Vaginal examinations

## Abstract

The purpose of this study is to investigate the impact of intrapartum events, such as rupture of membranes and vaginal examinations, on the infant gut microbiome in twins born vaginally. This prospective longitudinal cohort study included pregnant women with twin pregnancies beyond 36 weeks of gestation undergoing vaginal births. Stool samples were collected from newborns on days 4 and 28 for gut microbiome analysis using 16S rDNA sequencing. The infants were followed for at least 1 year. Forty babies were followed in this study. Gestational age at birth was 37.07 (36.75–37.57) weeks. Significant differences were observed between the first and second twins in the number of vaginal examinations (5.50 (4.00–7.25) vs. 1.00 (1.00–1.00), *P* < 0.001) and the rupture of membranes to birth time (524 (324–734.5) vs. 7.5 (4.5–9.0) minutes, *P* < 0.001). *Bifidobacterium* spp. were present in all samples, with differing relative abundance profiles on day 4 and day 28. On day 4, the first twins had over 50% *Bifidobacterium* spp., while second twins had less than 25%. By day 28, 75% of samples showed similar *Bifidobacterium* spp. profiles. Microbiome changes over time were more significant in second twins. At the species level, 20–40% of samples exhibited significant *Bifidobacterium longum* on day 4, higher in first twins compared to second twins.

*Conclusion*: Differences in the microbiome and its evolution between first and second twins were noted within the first 4 and 28 days of life, influenced by membrane rupture duration and the number of vaginal examinations for the first twin.

**Table:** 

**What is Known:** •*Differences in the gut microbiome have been shown between infants born via cesarean and vaginal birth.* •*Colonization by maternal microbiota at birth is influenced by multiple perinatal factors.* **What is New:** •*Between twins born vaginally, the gut microbiome differs based on the duration of membrane rupture and the number of vaginal examinations.* •*This difference is noticeable on day 4 of life but ceases to be noticeable by day 28.*

**Supplementary Information:**

The online version contains supplementary material available at 10.1007/s00431-025-06336-w.

## Introduction

The gut microbiota plays a significant role in maintaining overall health, particularly in immunity. Additionally, small changes early in life are associated with the development of numerous diseases such as asthma, allergies, inflammatory bowel disease, and obesity [1, 2]. In general, it is considered that the fetal gut is sterile, and that colonization begins upon exposure to bacteria at birth. However, some studies suggest that intestinal colonization begins in utero [3].

The mode of birth and the use of antibiotics during a cesarean or vaginal birth are believed to strongly influence neonatal microbial colonization [1, 4, 5]. It appears that babies born by cesarean birth have intestinal colonization that is significantly different and suboptimal to those born vaginally, and this is believed to impact immune dysregulation later in life [6]. This difference is attributed to the lack of contact with the maternal vaginal and fecal microbiota, as well as the perineal skin. The differences observed early in life based on the mode of birth diminish over time [4].

Current knowledge about the differences in gut microbiota has led to the consideration of procedures like vaginal seeding in cesarean births [7]. However, other factors can significantly influence microbial colonization, such as invasive procedures or vaginal examinations. As early as 1998, it was shown that the risk of chorioamnionitis significantly increased after seven vaginal examinations in women with premature rupture of membranes [8].

The present study arises from the hypothesis that the colonization of infants’ gut microbiota is likely to differ based on events during childbirth. Our aim was to evaluate the influence of rupture of membranes (ROM) and vaginal examinations on infants’ gut microbiome. To accomplish this, we examined twin pregnancies resulting in vaginal birth of both twins.

## Materials and methods

This is a prospective longitudinal cohort study that included pregnant women and babies after twin pregnancies greater than or equal to 36 weeks, with the first twin in cephalic presentation, who underwent a twin vaginal birth at a tertiary referral center. The study was conducted between February 2021 and January 2024. Written informed consent was obtained from the women agreeing to participate in the study, which was approved by the local Clinical Research Ethics Committee in August 2020, reference number PI-4264. All methods were conducted in accordance with the ethical standards of the declaration of Helsinki.

After obtaining written informed consent, maternal history and demographics were recorded from the woman’s medical records. Eligible women were assisted during childbirth following the local current protocol for twin births. Subsequently, stool samples of the babies were collected from the newborns on the 4th and 28th day of life to analyze the gut microbiome. The babies were followed up for at least 1 year after the birth of the last babies participating in the study.

Each participant had to meet all the following inclusion criteria: (a) a twin pregnancy greater than or equal to 36 weeks of gestation, (b) the first twin in cephalic presentation, (c) adequate prenatal care since the first trimester, (d) absence of diagnoses of fetal diseases or fetal growth abnormalities, (e) women over 18 years old with a good understanding of Spanish who provided written informed consent.

Families were excluded from participating or continuing in the study if (a) the woman was not mentally capable of understanding the information sheet and informed consent, (b) any criteria for active infection or chorioamnionitis was present, (c) the birth of either twin was not vaginal, (d) there was separation of one twin from the other during the first 4 days of life (e.g., admission of one of the twins), (e) antibiotics were administered to one of the twins during the first 4 days of life, or (f) the type of feeding was not the same for both twins during the first 4 days of life.

Demographic, pregnancy, birth, and children’s data were recorded. Data recorded included maternal age, maternal Body Mass Index (BMI), gestational age, chorionicity, diet, country of birth, race, medication, food supplements, time of ROM, type of vaginal birth, number, and timing of vaginal examinations, maximum temperature during labor, use of antibiotics during labor, neonatal weight, sex assigned at birth, Apgar 1/5, umbilical cord pH, any intervention by the neonatologists, neonatal length of stay in days, type of feeding until day 4, type of feeding until day 28, any incident requiring medical evaluation at day 4, at 1 week, at 2 weeks, at 3 weeks, at 4 weeks, and during the follow-up, children’s weight percentile using WHO child growth standards at the end of the study, any treatments, allergies,or health issues during the study.

Stool samples were collected on the fourth and twenty-eighth days after birth. Metagenomic analyses were performed at Probisearch SLU (Tres Cantos, Spain). To identify microbial diversity in infant fecal samples, the 16S rDNA gene was amplified using primers that flanked the variable regions.

V3 and V4. The PCR primer sequences were V3V4-CS1 (ACACTGACGACATGGTTCTACACCTACGGGNGGCWGCAG).

and V3V4-CS2.

(TACGGTAGCAGAGACTTGGTCTGACTACHVGGGTATCTAATCC), and the amplicons were sequenced using Illumina’s 2 × 250 bp paired-end technology. Resulting reads of quality controls were assembled and taxonomically classified by comparison with Greengenes database using a Bayesian classification method and a level of similarity of at least 97%. The diversity of the fecal microbiota was determined by calculating the Shannon–Weaver diversity index, considering the number and evenness of bacterial species, and the Bray–Curtis beta diversity index, which reflects the turnover of bacterial species among the different groups studied. The abundance of each genus/species detected in the samples was calculated and all the genera/species below 0.05% of abundance were grouped into “Other” category.

A formal sample size calculation could not be performed as there were no relevant data in the literature regarding the influence of ROM and vaginal exams on infant gut microbiome. We chose a sample size based on the study conducted by Dierikx et al. on the infant gut microbiome, which compared vaginal births with two groups of cesarean births that differed in the timing of intravenous antibiotic administration. In that study, 20 infants were used per group [9, 10]. Secondarily, for our study, our goal was to include 40 children: 20 first twins and 20 s twins.

### Statistical analysis

The distribution of the variables was assessed using the Shapiro–Wilk test and visual examination of histograms. Study variables were presented as means (95% confidence interval [CI]), or median (1st quartile–3rd quartile) for continuous variables. Absolute and relative frequencies were used for qualitative variables. Group comparisons were conducted using the ANOVA test, Kruskal–Wallis test, Mann–Whitney *U* test, two-tailed *χ*^2^-test, or two-tailed Fisher’s exact test, as appropriate. If results arise, statistical signification subsequent Tukey HSD or Nemenyi test was used as pairwise comparison, applying multiplicity correction methods. Bacterial genera detected in fecal samples were further analyzed using exploratory multivariate analyses such as Principal Component Analysis (PCA) and relationships between clinical features and microbiological data were evaluated using Multiple Factor Analysis (MFA). The significance level was set at 0.05.

## Results

Participation in the study was offered to 35 birthing people (all identifying themselves as women and mothers), and none of the eligible women refused. Twenty women (forty babies) met the criteria to complete the study. The 15 births that were excluded were due to cesarean section births or the admission of one of the twins after birth.

Of the 20 mothers who completed the study, maternal age was 36.50 (30.00–38.75) years and BMI was 25.48 (23.46–27.62) kg/m^2^. Gestational age at birth was 37.07 (36.75–37.57) weeks. There were 13 (65%) women with dichorionic pregnancies and 7 (35%) with monochorionic pregnancies. None of the women followed a restricted diet, was vegetarian, or had any food allergies; all reported following a standard diet. Seventeen (85%) women were European, 2 (10%) were Asian, and 1 (5%) was Latin American. Nineteen (95%) used dietary supplements for twin pregnancy, and none of them probiotics. During pregnancy, 14 (70%) women used oral iron, 6 (30%) levothyroxine, 10 (50%) cholecalciferol, 1 (5%) 100 mg of acetylsalicylic acid, and 2 (10%) enoxaparin.

Regarding labor, all of them used epidural analgesia. Antibiotics were used before birth in 7 (35%) of the women. Penicillin was administered to 4 (20%) women for a positive vaginal culture for group B streptococcus, and ampicillin and gentamicin were administered to 3 (15%) women for ROM of the first twin for more than 12 h. The maximum temperature during labor was 36.35 (35.72–37.37) degrees Celsius; in none of them, the maximum temperature exceeded 37.8 degrees Celsius (100 degrees Fahrenheit).

There were statistically significant differences between the first and the second twin in the number of vaginal examinations (5.50 (4.00–7.25) vs. 1.00 (1.00–1.00), *P* < 0.001), the number of vaginal examinations with ROM (4.00 (3.00–5.00) vs. 0.00 (0.00–0.00), *P* < 0.001), and the ROM to birth time (524 (324–734.5) vs 7.5 (4.5–9.0) min, *P* < 0.001). There were no statistically significant differences for the other parameters studied (Table 1).

**Table 1:** Labor and child features for the first and second twin

	First twin	Second twin	
Characteristics	(*N* = 20)	(*N* = 20)	*P* value
Vaginal examinations,			< 0.001
Median (Q1–Q3)	5.50 (4.00–7.25)	1.00 (1.00–1.00)	
Vaginal examinations with ROM,			< 0.001
Median (Q1–Q3)	4.00 (3.00–5.00)	0.00 (0.00–0.00)	
Admission to birth time,			0.725
Median (Q1–Q3)	736.50 (565.50–887.50)	741.50 (575.75–892.75)	
ROM to birth time,			< 0.001
Median (Q1–Q3)	524.00 (348.50–714.75)	7.50 (4.75–9.00)	
Labor type,			0.211
Natural	14 (70.0)	15 (75.0)	
Forceps	3 (15.0)	0 (0)	
Suction cup	3 (15.0)	3 (15.0)	
Breech extraction	0 (0)	2 (10.0)	
Resuscitation type,			0.797
No	18 (90.0)	16 (80.0)	
Positive pressure	1 (5.0)	1 (5.0)	
Warming and stimulation	1 (5.0)	3 (15.0)	
Neonatal weight (g),			0.685
Median (Q1–Q3)	2552.50 (2202.50–2735.00)	2515.00 (2363.75–2775.25)	
Apgar 1′,			0.505
Median (Q1–Q3)	9.00 (9.00–9.00)	9.00 (8.75–9.00)	
Apgar 5′,			1
Median (Q1–Q3)	10.00 (9.00–10.00)	10.00 (9.00–10.00)	
Umbilical cord pH,			0.095
Median (Q1–Q3)	7.30 (7.27–7.33)	7.27 (7.22–7.32)	
Days in NICU			1
Median (Q1–Q3)	0.00 (0.00–0.00)	0.00 (0.00–0.00)	
Days until discharge			1
Median (Q1–Q3)	2.00 (2.00–3.00)	2.00 (2.00–3.00)	
Sex assigned at birth			1
Female	10 (50.0)	11 (55.0)	
Male	10 (50.0)	9 (45.0)	
Type of feeding until day 4			1
Breastfed	10 (50.0)	10 (50.0)	
Formula	1 (5.0)	1 (5.0)	
Mixed	9 (45.0)	9 (45.0)	
Type of feeding until day 28			1
Breastfed	13 (65.0)	13 (65.0)	
Formula	1 (5.0)	1 (5.0)	
Mixed	6 (30.0)	6 (30.0)	
Weight percentile at the end of the study^a^			0.881
Median (Q1–Q3)	65.50 (45.03–70.85)	65.50 (48.03–77.88)	

Abbreviations: *ROM*, rupture of membranes; *NICU*, neonatal intensive care unit

Data are presented as number (percentage) unless otherwise specified

The Kruskal–Wallis and Fisher tests were used to assess for differences by order of birth for each variable

^a^Weight percentile calculated using WHO child growth standards

Concerning postnatal follow-up, the infants were followed for 25.5 (19.0–30.0) months. The growth of the 40 infants was adequate according to WHO growth charts (Table 1). Four (20%) second twins experienced viral infections requiring treatment during follow-up compared to 2 (10%) first twins, *P* = 0.661 (infections occurred between 1 and 18 months of age and were caused by respiratory syncytial virus in five children, with one child having no etiological diagnosis). One first twin was diagnosed with atopic dermatitis and one second twin with psoriasis at 13 months of age. During follow-up, three surgeries were performed: one myringotomy and one inguinal hernia repair among the first twins, and one dacryocystorhinostomy in a second twin. Currently, two second twins are undergoing treatment with budesonide, while one first twin is on budesonide and another on dexchlorpheniramine.

Regarding the microbiome, results were obtained from the analysis of 79 out of the 80 stool samples. The sample from which results were not obtained corresponded to a day 4 sample from a second twin. No significant differences were found in the alpha and beta diversity indices analyzed at the phylum, genus, and species levels (Table 2).

**Table 2:** Alpha and beta diversity at different taxonomic levels of fecal samples from twin pairs, by birth order and days of life

	Day 4th		Day 28th		
	First twin	Second twin	First twin	Second twin	***P*** value^a^
Shannon index					
Phylum	0.318 (0.242–0.394)	0.334 (0.257–0.411)	0.337 (0.272–0.401)	0.372 (0.319–0.425)	0.683
Genus	0.586 (0.468–0.705)	0.575 (0.435–0.716)	0.520 (0.427–0.614)	0.614 (0.526–0.703)	0.646
Species	0.697 (0.548–0.847)	0.718 (0.563–0.873)	0.683 (0.564–0.802)	0.779 (0.681–0.878)	0.712
Bray–Curtis distance					
Phylum	0.443 (0.369–0.517)	0.411 (0.337–0.486)	0,444 (0.385–0.503)	0.365 (0.279–0.451)	0.345
Genus	0.582 (0.544–0.619)	0.577 (0.534–0.619)	0.565 (0.526–0.605)	0.551 (0.499–0.603)	0.732
Species	0.629 (0.599–0.659)	0.630 (0.599–0.660)	0.620 (0.592–0.648)	0.611 (0.579–0.642)	0.753

Data are presented as mean (95% CI)

^a^ANOVA test

**Fig. 1:**
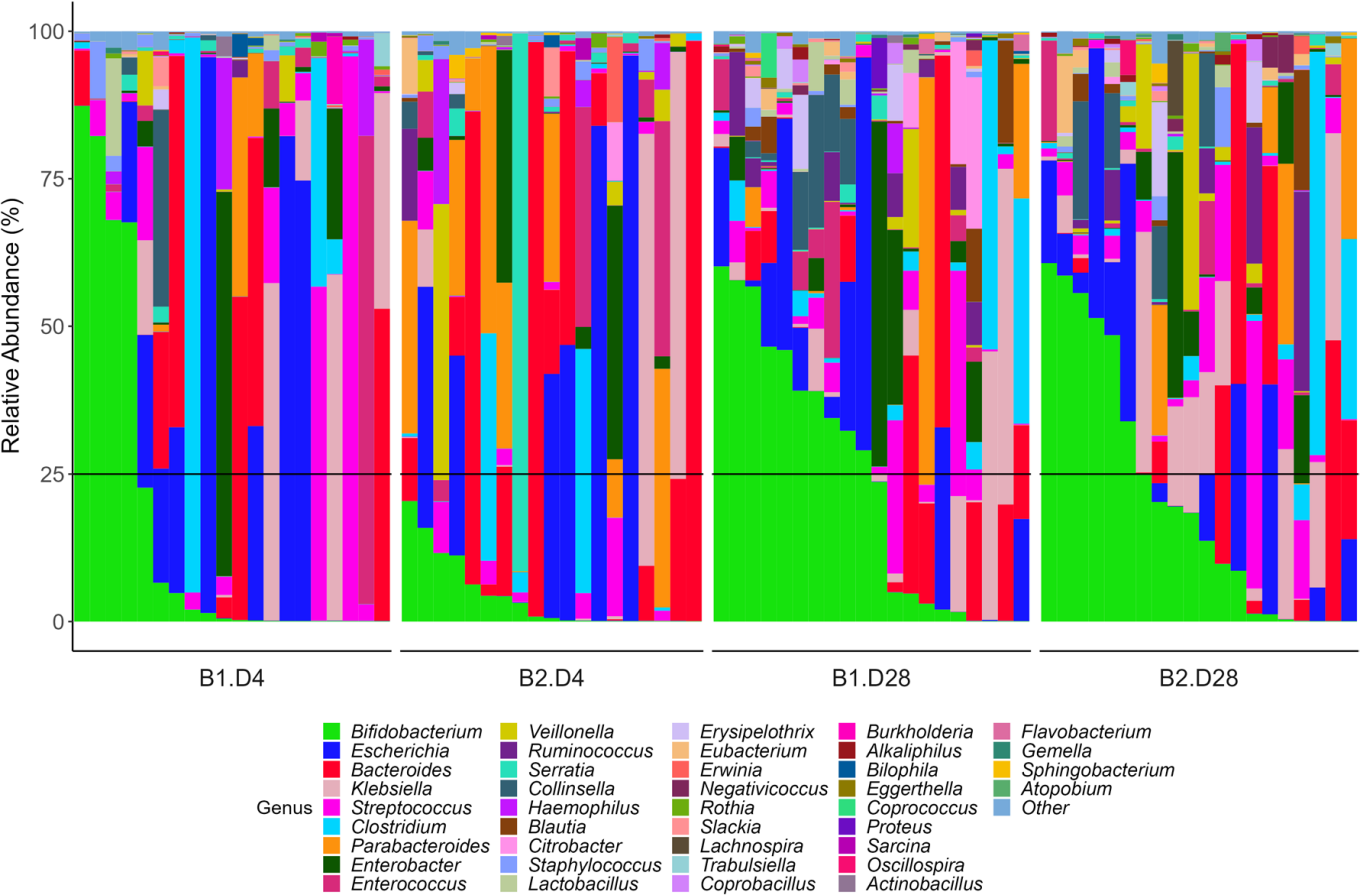
Relative abundance at genus level of fecal samples collected from twins at day 4 and 28 after birth (cut-off of 0.05%). Subjects are sorted from higher to lower relative abundance of the main genus (*Bifidobacterium*), order of birth and collecting time. Black horizontal line is 25% relative abundance. B1, first twin; B2, second twin; D4, day 4; D28, day 28

At genus level, *Bifidobacterium* was present in all samples, although the relative abundance profile differed on day 4 and day 28. On day 4, only 50% of the samples have appreciable quantities. Furthermore, the relative abundance of *Bifidobacterium* spp. in the first twins was higher (more than 50%) on day 4 than in the second twins (where an abundance of 25% of *Bifidobacterium* spp. was never reached). By day 28, 75% of the samples exhibited appreciable quantities of *Bifidobacterium* spp., with a similar profile for first and second twins (Fig. 1).

**Fig. 2:**
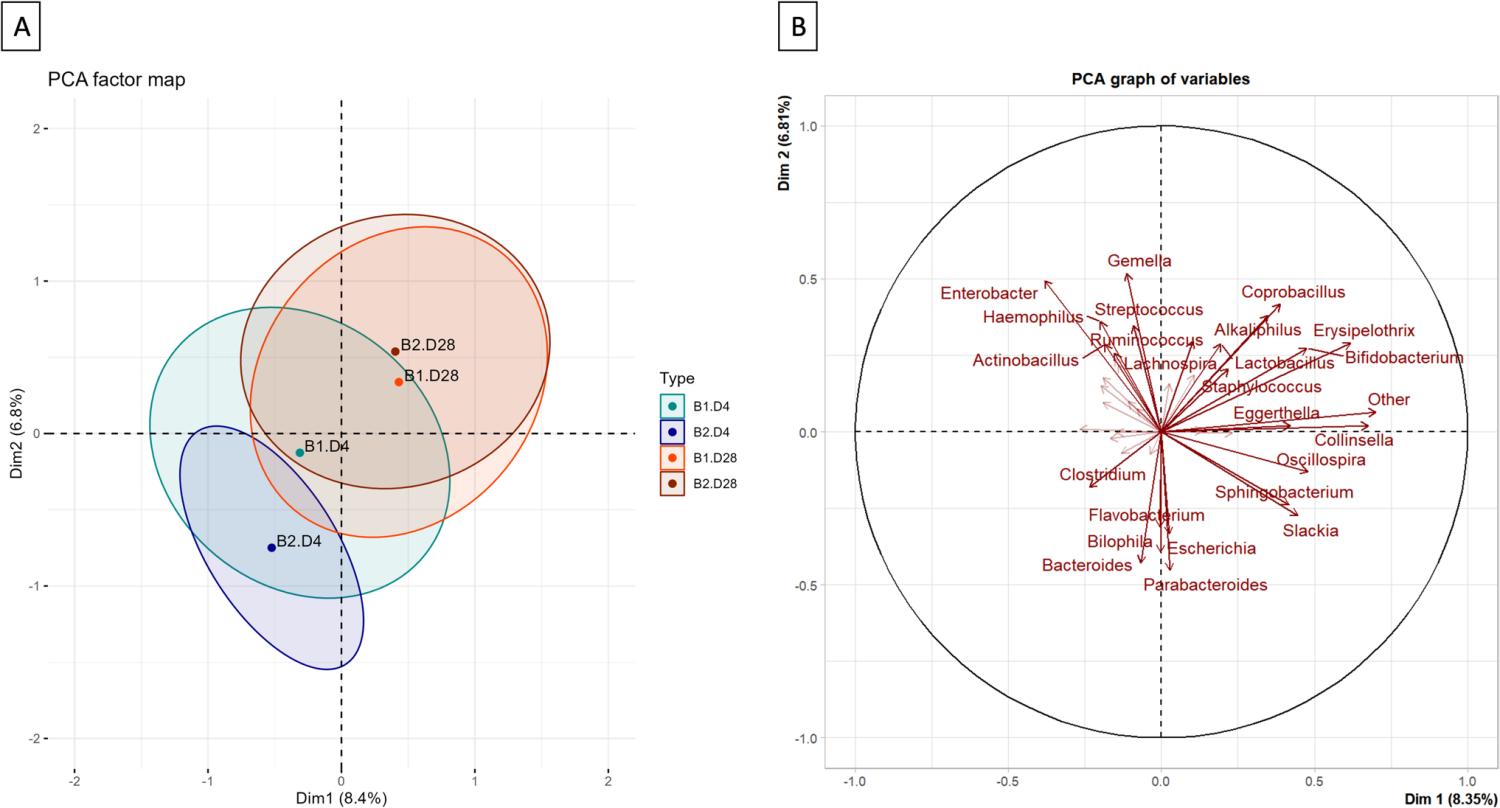
PCA chart of variables where the first 25 contributive variables are plotted with continuous arrows and labelled, and all the others are plotted with dashed arrows (**A**); PCA chart of the 90% confidence ellipses by order of birth and day of sample collection (**B**) for the first two dimensions of the relative abundance data at the genus level (cut-off 0.05%); B1D4, first twin day 4 samples; B2D4, second twin day 4 samples; B1D28, first twin day 28 samples; B2D28, second twin day 28 samples

The Principal Component Analysis (PCA) was performed with the 41 quantitative variables retained after applying a 0.05% cut-off at the genus level (40 genera + 1 “others”). Regarding the contribution of the most abundant genera, the genus *Bifidobacterium* contributes the most to the first dimension or component (ctr = 6.6) and *Enterobacter* contributes the most to the second dimension or component (ctr = 8.7). The contribution of the different genera is shown in Fig. 2 where the 1 st and 2nd dimensions explain the fifteen percent of the variability of the data (Fig. 2A).

In the 90% confidence ellipses of the individuals chart, a change in the microbiota at the genus level over time could be seen (Fig. 2B). This change was greater and significant in second twins. A greater homogeneity was also observed at day 4 in the second twins. These differences disappeared after 28 days from birth, due to an increase in the relative abundance of *Bifidobacterium* spp. in addition to other genera (Fig. 2B).

**Fig. 3:**
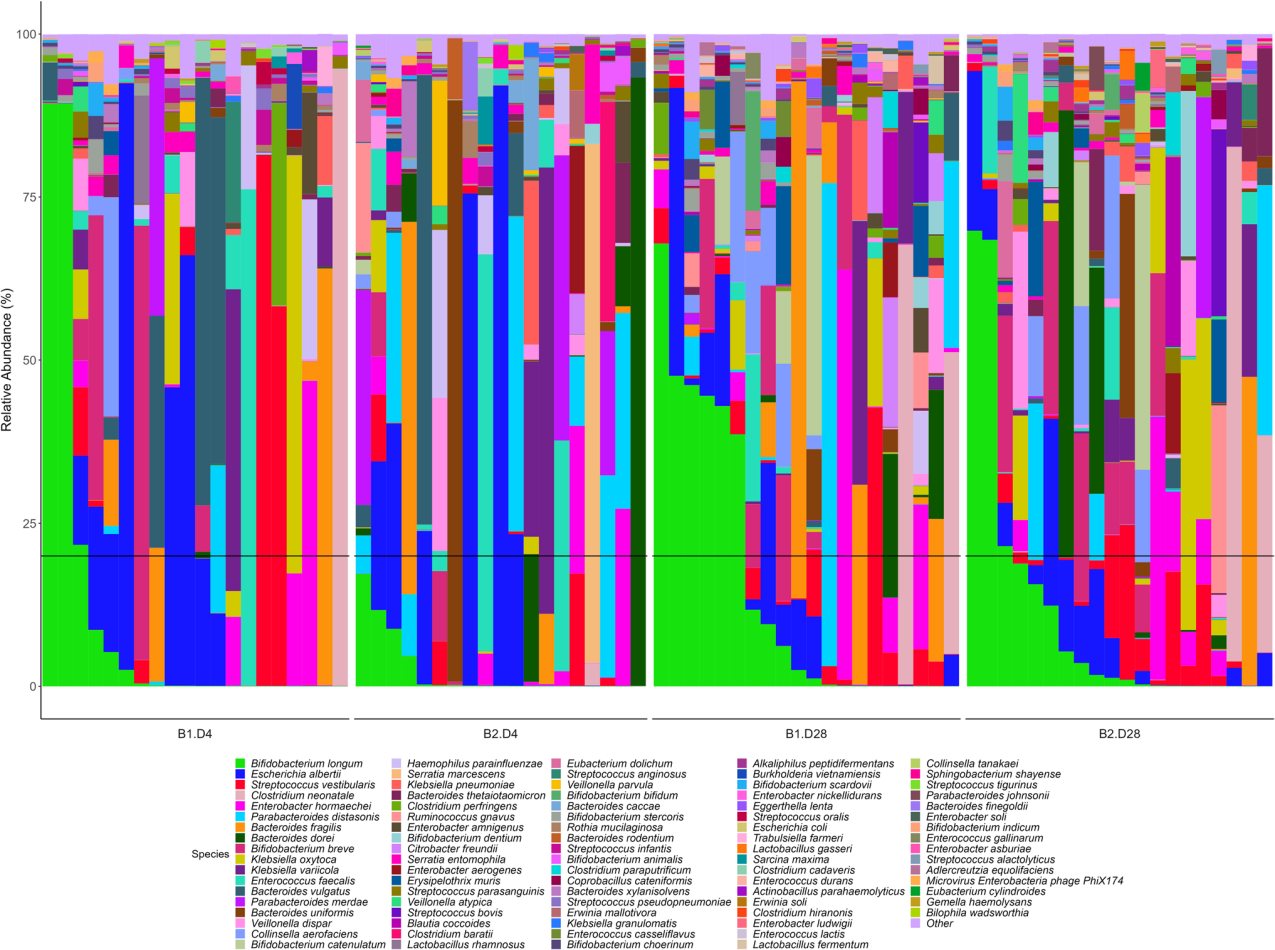
Relative abundance at species level of fecal samples collected from twins at days 4 and 28 after birth (cut-off of 0.05%). Subjects are sorted from higher to lower relative abundance of the main species (*Bifidobacterium longum*), order of birth and collecting time. Black horizontal line is 20% relative abundance. B1, first twin; B2, second twin; D4, day 4; D28, day 28

At the species level, the profile of relative abundances for the major species indicates that between 20 and 40% of the samples exhibited significant quantities of *Bifidobacterium longum* on day 4. This presence was higher in first twins compared to second twins (exceeding 80% in some cases for first twins, while in second twins it did not exceed 20% at this sampling time) (Fig. 3).

**Fig. 4:**
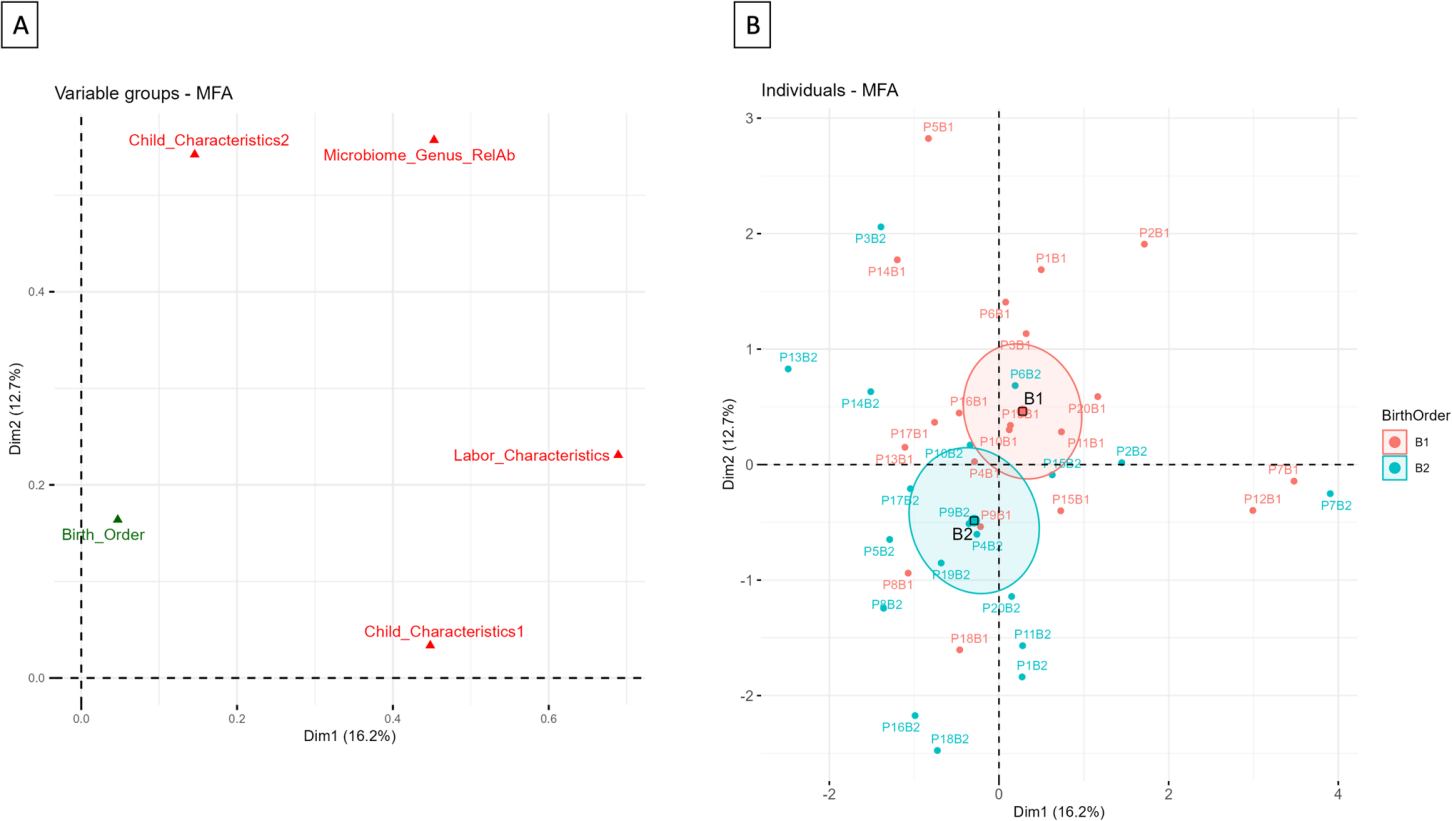
MFA chart of the groups of variables where the groups of variables used in the analysis appear in red and the supplementary qualitative variable appear in green in the coordinates position assigned for the two first dimensions (**A**); MFA chart of the individuals stratified by order of birth; the first twins were plotted in red and the second twins were plotted in green. The centroid of each stratification level with the 95% confidence ellipse were also plotted (**B**). B1, first twin; B2, second twin

The relationships between the relative abundance microbiome data at genus level (after cut-off 0.05%) and clinical features related with labor and children were evaluated by Multiple Factor Analysis (MFA). To do that, clinical features were divided into three groups: labor characteristics, child quantitative characteristics, and child qualitative characteristics (see Suppl Table 1). Further, the order of birth was used as supplementary qualitative variable. The first two dimensions of the MFA explain the 30% of the variability with all the groups of variables located in the first square of the cartesian axes, showing a positive relationship between them. Further, labor features and microbiome data are the most contributory to the 1 st dimension, while the microbiome data and child qualitative features are the most contributory to the 2nd dimension (Fig. 4A). Regarding individuals map, the 95% confidence ellipse stratified by order of birth was separated, and this takes place in the 2nd dimension (Fig. 4B). The main contributory labor features were time from admission to birth, the duration of ROM, total vaginal examinations with ROM, and vaginal examinations with ROM which shown good correlation (positive or negative) with genera detected in the fecal samples of the twins at day 4 like *Escherichia*, *Klebsiella*, or *Clostridium* in the 1 st dimension and *Bacteroides*, *Streptococcus*, and *Bifidobacterium* in the 2nd dimension (see details of the coordinates and contribution of the mapping variables to the 1 st and 2nd dimensions of the MFA in Suppl. Materials Suppl. Table 1 and Suppl. Figure 1 A, B, C, and Suppl. Figure 2 A, B.)

## Discussion

Our study showed differences in the gut microbiome between twins born vaginally on day 4 of life. By focusing on twins who received identical postnatal treatment, remained together, and were both born vaginally, we identified the primary factors influencing these differences as the number of vaginal examinations with ROM and the duration of ROM. By day 28, as both twins shared the same environment with similar exposure to environmental factors and feeding, colonization by the *Bifidobacterium* genus became evident, and there was a clear trend toward microbiome homogenization.

Changes in the microbiome based on the duration of ROM have been recently evaluated in the MATER study [11]. This study evaluates whether the duration of ROM can cause the changes in colonization that occur during childbirth. Using a vaginal sample from each participant, this study examines differences in the maternal vaginal microbiome in relation to the duration of ROM and proposes artificial membrane rupture, immediately prior to delivery so the duration of ROM is short, as an alternative to vaginal seeding in elective cesarean births. However, the number of vaginal examinations by obstetricians and their potential influence were not analyzed in the study.

Regarding vaginal examinations, there is no clear evidence linking their use to alterations in the microbiome [12]. However, the number of examinations performed has been associated with febrile conditions and clinical chorioamnionitis [13, 14]. This increase in febrile episodes and diagnoses of chorioamnionitis related to the number of vaginal examinations suggests that these may influence the microbiota. Unlike the duration of ROM, which is a factor that is difficult to modify or avoid without interfering with the birth process, the frequency of vaginal examinations is a modifiable factor. In this context, intrapartum ultrasound has emerged as a complementary technique that may assist in reducing the number of vaginal examinations when appropriate [15–17]. Intrapartum ultrasound monitoring has been shown to result in lower rates of clinical and histopathological chorioamnionitis in cases of PROM and has been demonstrated to be feasible in labor inductions [18, 19].

Concerning the follow-up outcomes of the infants, we did not observe any differences between the first and second twins in any of the studied health parameters. There are previous reports showing greater risks for the second twins, although these are generally associated with complications during birth and neither with the mode of birth, nor with differences in the duration of ROM or the number of vaginal examinations [20, 21]. Moreover, considering the findings of the Twin Birth Study, which revealed no significant differences justifying a policy of scheduled cesarean sections in twin pregnancies, and recognizing the numerous risks associated with cesarean births in singleton pregnancies (potentially influenced by changes in the microbiome), we believe that the differences observed in our study would not justify a change in clinical practice regarding the mode of birth [22–28].

### Strengths and limitations

The primary strength of this study lies in the control of potential confounding factors, achieved by focusing on twin pregnancies and employing strict selection criteria. By excluding cases where twins were separated at birth for any reason or did not receive the same feeding regimen, we ensured that the infants were analyzed in precisely the same environment.

The main limitation is the lack of analysis of maternal vaginal samples. Although the women who participated in the study were healthy and maintained twin pregnancies beyond 36 weeks, we believe that studying vaginal samples before and after the first birth would have allowed us to determine if the birth of the first baby could influence the maternal microbiome. Nonetheless, we believe that such an influence is likely to be limited.

Another limitation lies in the relatively small sample size. Although the twin-based design provided an opportunity to explore differences in gut microbiome colonization under strictly controlled conditions, it did not allow for a robust analysis of other relevant perinatal factors, such as intrapartum antibiotic exposure or differences in neonatal feeding practices, since these factors affected both twins equally.

## Conclusion

There are differences in the microbiome and its evolution within the first 4 and 28 days of life between the first and second twins born via vaginal birth. The primary factors that may contribute to these differences are the duration of ROM and the number of vaginal examinations conducted with ruptured membranes for the first twin. The observed differences indicate a lesser dispersion of the microbiome in the second twin at day 4, allowing for a clear differentiation of the microbiome by day 28 of life. In contrast, this differentiation is not apparent in the first twin. Therefore, in twins who share the same environment and mother, not all vaginal births are equal. Although the gut microbiome appears to progressively homogenize when similar nurturing and environmental conditions are met, clear differences exist in the early days of life.

## Supplementary Information

Below is the link to the electronic supplementary material.

ESM 1 (PNG 557 KB)

High Resolution Image (TIFF 25.7 MB)

ESM 1 (PNG 227 KB)

High Resolution Image (TIFF 25.7 MB)

ESM 3 (DOCX 19.1 KB)

## Data Availability

The data that support the findings of this study are not openly available due to reasons of sensitivity and are available from the corresponding author upon reasonable request.

## Abbreviations

BMI: Body Mass Index
MFA: Multiple Factor Analysis
ROM: Rupture of membranes
PCA: Principal Component Analysis
