# Supplementary figures and images for: Gut microbiome differences after vaginal birth in relation to rupture of membranes at term: a prospective longitudinal cohort study of twins
Source: Eur J Pediatr. 2025 Jul 30;184(8):511. doi: 10.1007/s00431-025-06336-w (PMC12310857; doi:10.1007/s00431-025-06336-w)

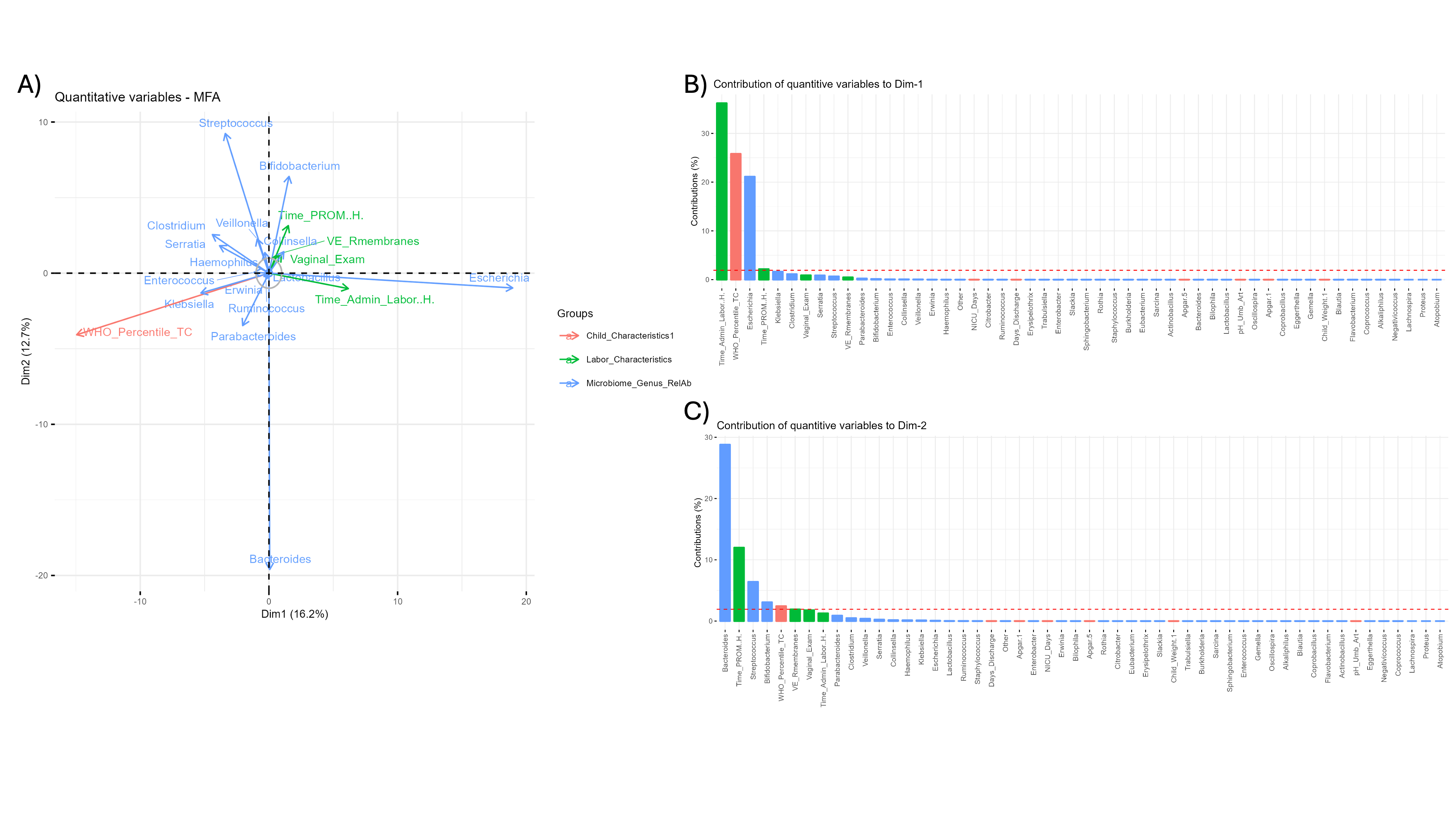

Supplement: Supplementary file 1 — (PNG 557 KB) [file 431_2025_6336_Fig5_ESM.png]

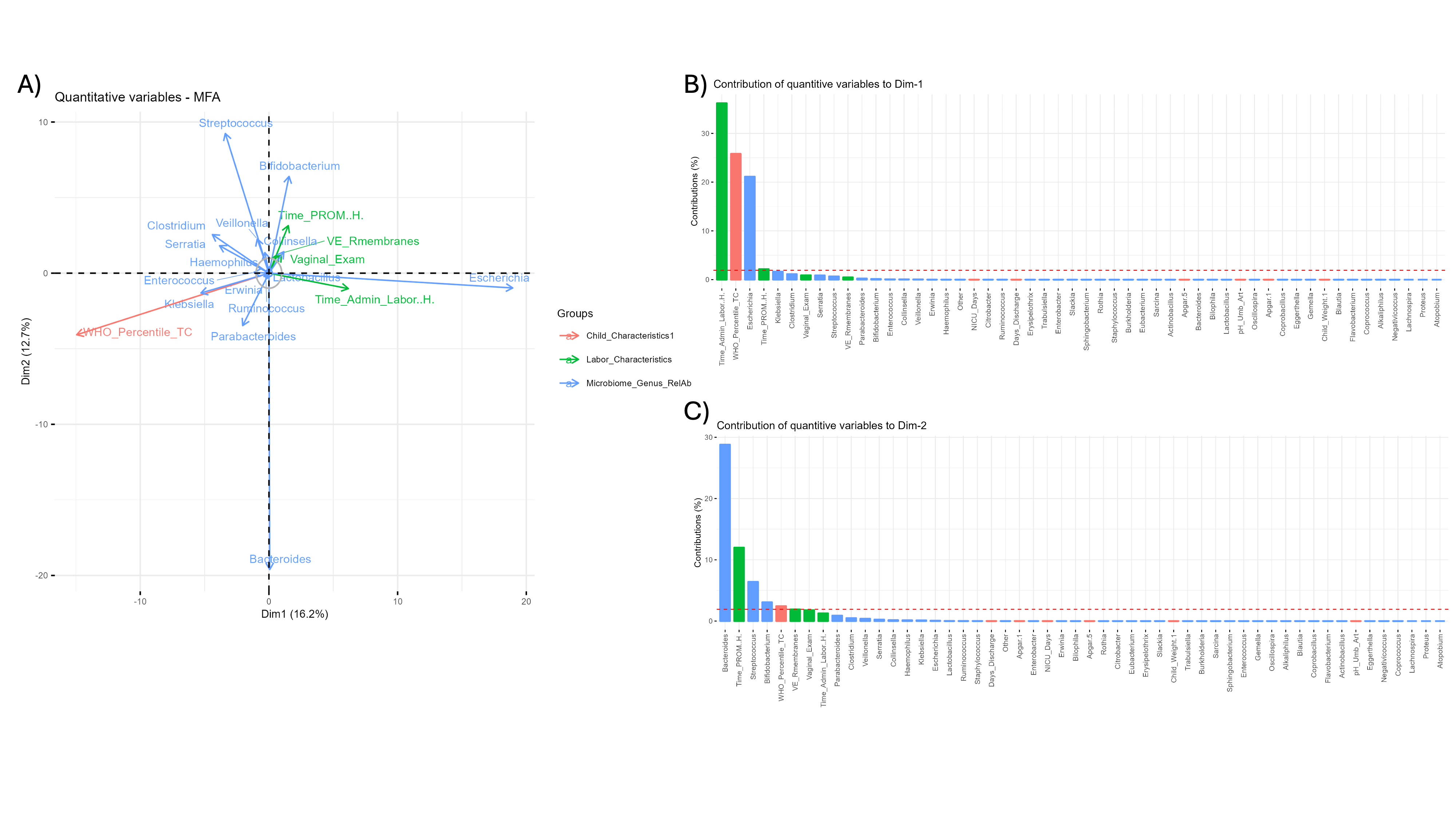

Supplement: Supplementary file 2 — High Resolution Image (TIFF 25.7 MB) [file 431_2025_6336_MOESM1_ESM.tiff]

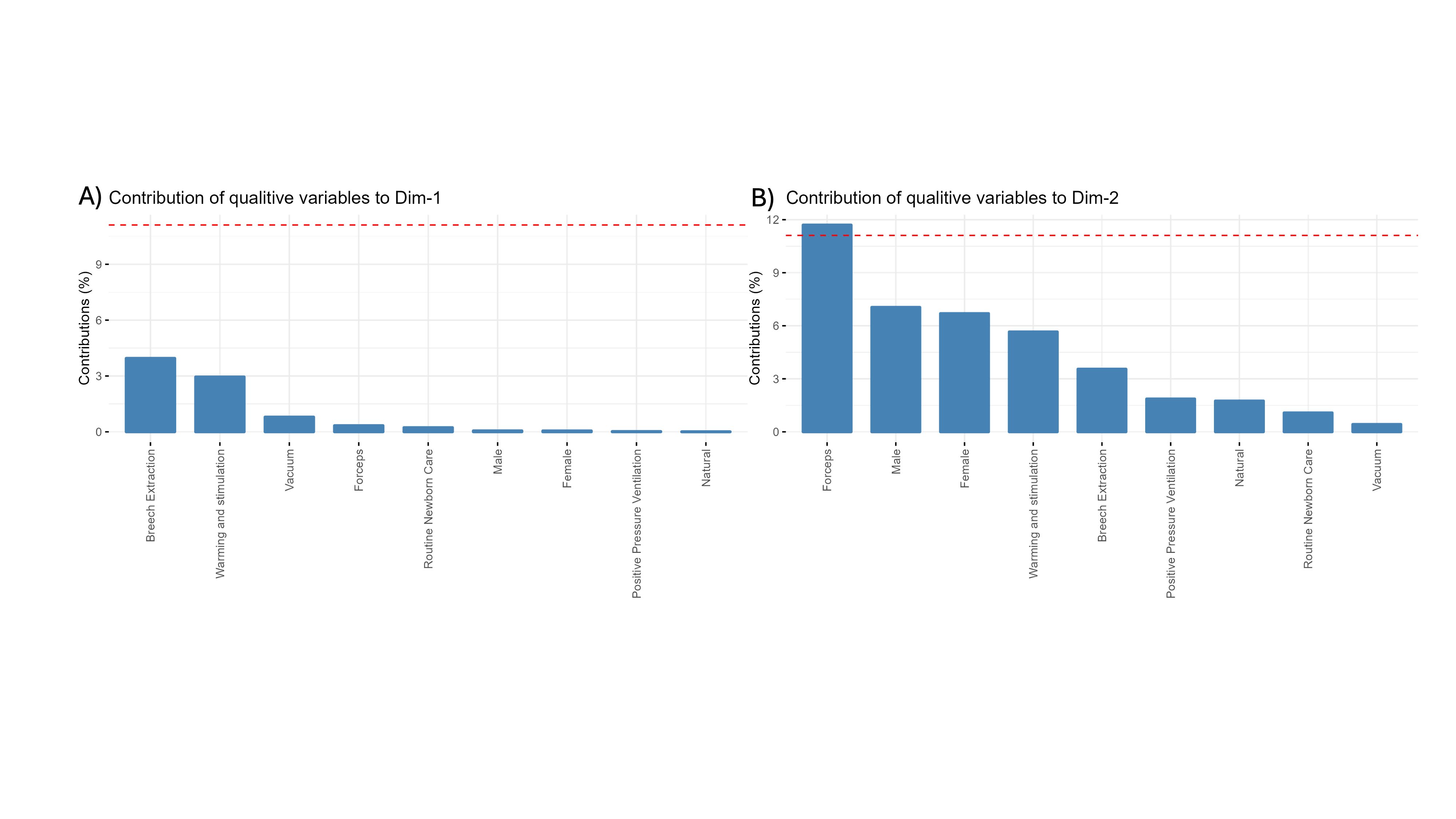

Supplement: Supplementary file 3 — (PNG 227 KB) [file 431_2025_6336_Fig6_ESM.png]

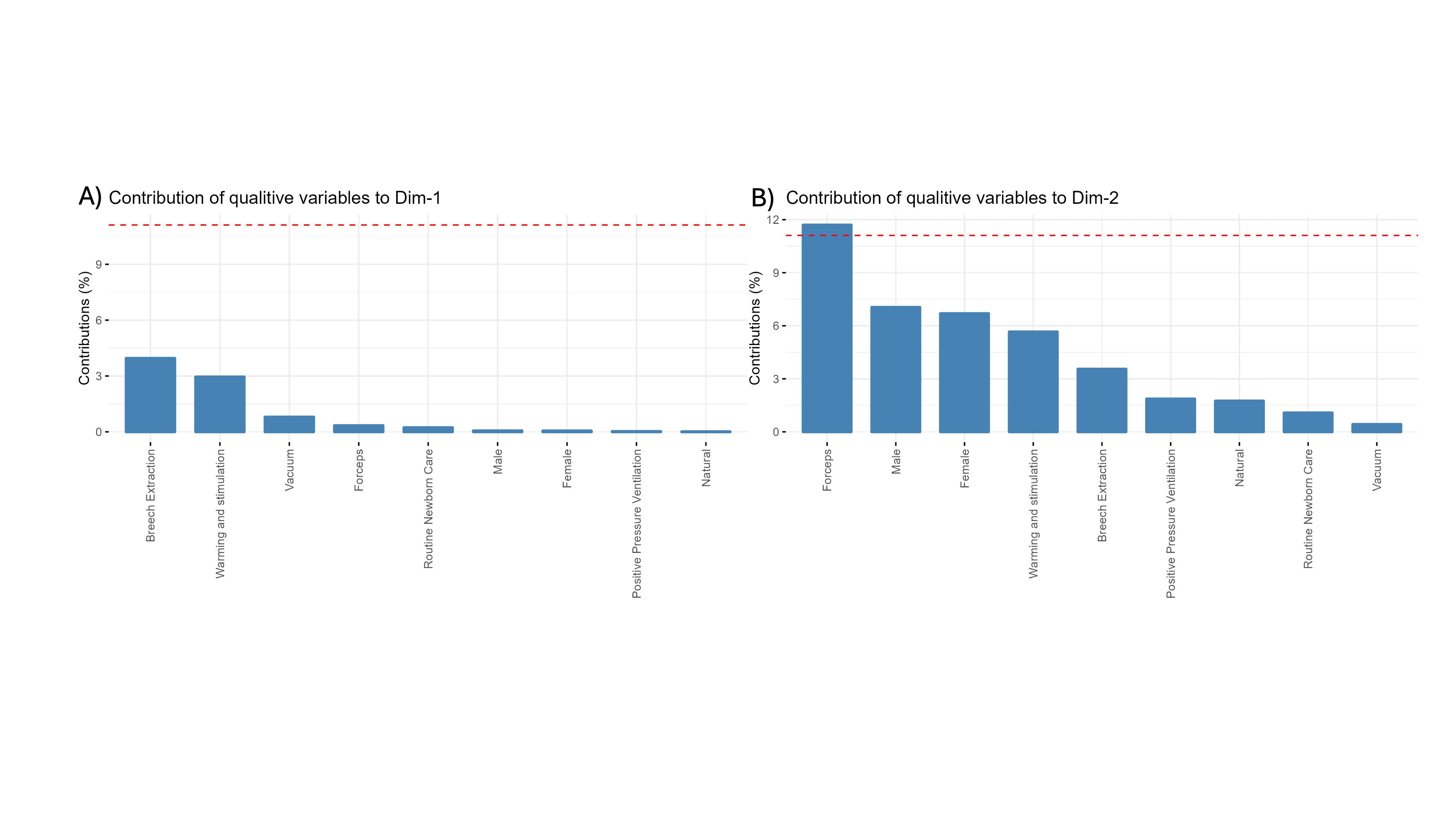

Supplement: Supplementary file 4 — High Resolution Image (TIFF 25.7 MB) [file 431_2025_6336_MOESM2_ESM.tiff]
