# Supplementary material for: Gut microbiome differences after vaginal birth in relation to rupture of membranes at term: a prospective longitudinal cohort study of twins
Source: Eur J Pediatr. 2025 Jul 30;184(8):511. doi: 10.1007/s00431-025-06336-w (PMC12310857; doi:10.1007/s00431-025-06336-w)
Supplement: Supplementary file 5 — (DOCX 19.1 KB) [file 431_2025_6336_MOESM3_ESM.docx]

Supplementary information

**Suppl. Figure 1:** MFA chart of the 20 more contributive quantitative variables colored by group of variables (A); ordered bar chart of the contribution (%) of the quantitative variables colored by group of variables to the 1^st^ dimension of the MFA (B); ordered bar chart of the contribution (%) of the quantitative variables colored by group of variables to the 2^nd^ dimension of the MFA (C).

**Suppl. Figure 2:** Ordered bar chart of the contribution (%) of each of the levels of the qualitative variables included to the 1^st^ dimension of the MFA (A); ordered bar chart of the contribution (%) of each of the levels of the qualitative variables included to the 2^nd^ dimension of the MFA (B).

**Suppl. Table 1:** Labor and Child features evaluated in the Multiple Factor Analysis together with the relative abundance of the 40 genera detected in fecal samples after cutoff at 0.05% + the Other category at day 4. Coordinates and contribution to 1^st^ and 2^nd^ Dimensions.

| **Abbreviation** | **Feature name** | **Feature levels** | **Coordinates**  **Dim 1** | **Coordinates**  **Dim 2** | **Contribution**  **Dim 1 (%)** | **Contribution**  **Dim 2 (%)** |
| --- | --- | --- | --- | --- | --- | --- |
| **Labor_Characteristics** |  |  |  |  |  |  |
| Vaginal_Exam | Total vaginal examinations |  | 0.971 | 1209 | 0.89 | 1.77 |
| VE_Rmembranes | Vaginal examinations with ROM |  | 0.703 | 1256 | 0.47 | 1.91 |
| Time_Admin_Labor..H. | Admission to labor time (h) |  | 6171 | -1022 | 36.19 | 1.26 |
| Time_PROM..H. | Premature ROM time (h) |  | 1506 | 3149 | 2.16 | 11.99 |
| **Child_Characteristics1** |  |  |  |  |  |  |
| Child_Weight.1 | Neonatal weight (Kg) |  | -0.005 | -0.088 | 3.18e-06 | 1.14e-03 |
| Apgar.1 | Apgar 1’ |  | -0.009 | -0.191 | 1.02e-05 | 5.38e-03 |
| Apgar.5 | Apgar 5’ |  | 0.045 | -0.112 | 2.32e-04 | 1.83e-03 |
| pH_Umb_Art | Umbilical Cord pH |  | 0.020 | 0.002 | 4.61e-05 | 5.82e-07 |
| NICU_Days | Days at NICU |  | 0.190 | 0.173 | 4.17e-03 | 4.39e-03 |
| Days_Discharge | Days until discharge |  | -0.155 | 0.226 | 2.79e-03 | 7.54e-03 |
| WHO_Percentile_TC | Weight percentile at the end of the study |  | -14.941 | -4077 | 25.80 | 2.45 |
| **Child_Characteristics2** |  |  |  |  |  |  |
| Sex | Sex assigned at birth | Female | 0.071 | 0.627 | 5.24e-03 | 6.69 |
|  |  | Male | -0.074 | -0.660 | 0.05 | 7.04 |
| Labor_Type | Obstetrician intervention at labor | Forceps | 0.460 | 2140 | 0.33 | 11.70 |
|  |  | Breech Extraction | -1938 | -1443 | 395 | 3.55 |
|  |  | Natural | -0.018 | 0.271 | 4.99e-3 | 1.75 |
|  |  | Vacuum | 0.502 | 0.287 | 0.79 | 0.42 |
| Res_type | Neonatologist intervention at birth | Routine Newborn Care | 0.113 | -0.193 | 0.23 | 1.08 |
|  |  | Positive Pressure Ventilation | 0.135 | 1.046 | 0.02 | 1.86 |
|  |  | Warming and stimulation | 1.367 | 1.488 | 2.95 | 5.65 |
